# TAF8 regions important for TFIID lobe B assembly or for TAF2 interactions are required for embryonic stem cell survival

**DOI:** 10.1016/j.jbc.2021.101288

**Published:** 2021-10-09

**Authors:** Elisabeth Scheer, Jie Luo, Andrea Bernardini, Frank Ruffenach, Jean-Marie Garnier, Isabelle Kolb-Cheynel, Kapil Gupta, Imre Berger, Jeff Ranish, László Tora

**Affiliations:** 1 Institut de Génétique et de Biologie Moléculaire et Cellulaire (IGBMC), Centre National de la Recherche Scientifique, UMR7104, Institut National de la Santé et de la Recherche Médicale, U964, Université de Strasbourg, Illkirch, France; 2 Institute for Systems Biology (ISB), Seattle, Washington, USA; 3 School of Biochemistry and Bristol Research Centre for Synthetic Biology BrisSynBio, University of Bristol, Bristol, UK

**Keywords:** TAF8, TFIID, structure, function, TATA-binding protein (TBP), TBP-associated factors (TAFs), embryonic stem cells (ESCs), CRISPR/Cas9, knock out, viability, co-IP, coimmunoprecipitation, CXMS, cross-linking mass spectrometry, EM, electron microscopy, ESC, embryonic stem cell, FACS, fluorescence activated cell sorting, GTF, general transcription factor, HFD, histone fold domain, ID, intermediary domain, IP, immunoprecipitation, PIC, preinitiation complex, Pol II, polymerase II, PRD, proline-rich domain, SPR, surface plasmon resonance, TAF, TBP-associated factor, TBP, TATA-binding protein, WCE, whole cell extract

## Abstract

The human general transcription factor TFIID is composed of the TATA-binding protein (TBP) and 13 TBP-associated factors (TAFs). In eukaryotic cells, TFIID is thought to nucleate RNA polymerase II (Pol II) preinitiation complex formation on all protein coding gene promoters and thus, be crucial for Pol II transcription. TFIID is composed of three lobes, named A, B, and C. A 5TAF core complex can be assembled *in vitro* constituting a building block for the further assembly of either lobe A or B in TFIID. Structural studies showed that TAF8 forms a histone fold pair with TAF10 in lobe B and participates in connecting lobe B to lobe C. To better understand the role of TAF8 in TFIID, we have investigated the requirement of the different regions of TAF8 for the *in vitro* assembly of lobe B and C and the importance of certain TAF8 regions for mouse embryonic stem cell (ESC) viability. We have identified a region of TAF8 distinct from the histone fold domain important for assembling with the 5TAF core complex in lobe B. We also delineated four more regions of TAF8 each individually required for interacting with TAF2 in lobe C. Moreover, CRISPR/Cas9-mediated gene editing indicated that the 5TAF core-interacting TAF8 domain and the proline-rich domain of TAF8 that interacts with TAF2 are both required for mouse embryonic stem cell survival. Thus, our study defines distinct TAF8 regions involved in connecting TFIID lobe B to lobe C that appear crucial for TFIID function and consequent ESC survival.

Transcription regulators in eukaryotes can be divided into three functional classes: gene-specific transcription regulators, cofactor complexes, and the general RNA polymerase transcription machinery. Their collaborative action is necessary to access specific loci in chromatin and allow precise transcription initiation (1). Regulated RNA polymerase II (Pol II) transcription requires a highly concerted, stepwise assembly of transcription factor complexes that form the preinitiation complex (PIC). A functional PIC consists of Pol II and six general transcription factors (GTFs): TFIIA, TFIIB, TFIID, TFIIE, TFIIF, and TFIIH (2, 3). The evolutionary conserved TFIID complex is the first GTF that binds gene promoters and along with other GTFs, acts as a platform for PIC formation and consequent transcription initiation (3, 4). TFIID is a multisubunit complex of about 1 MDa, composed of the TATA box-binding protein (TBP) and 13 (14 in yeast) TBP-associated factors (TAFs) (5). TFIID not only is essential for the recognition of core promoter sequences and the recruitment of the PIC, but also involved in gene expression *via* its interactions with cofactors, gene-specific activators and repressors, and chromatin modifications associated with active regions of the genome (4, 6, 7).

Human TAF8 is a 310 amino acid protein harboring a histone fold domain (HFD) at its N-terminal end, which interacts with the HFD of TAF10, to form a noncanonical histone fold pair arrangement in TFIID (8, 9, 10). TAF8 also interacts with TAF2 and TAF2-TAF8-TAF10 subcomplex assembles in the cytoplasm of human cells (10). Biochemical studies revealed that TFIID is assembled in a stepwise manner, first forming a stable 5TAF core complex, consisting of two copies each of TAF5-TAF6-TAF9-TAF4-TAF12. On the one hand, this core is bound by the TAF8-TAF10 heterodimer, forming the 7TAF complex, similar to lobe B (11, 12), or by TAF8-TAF10-TAF2 complex, forming the 8TAF complex (10, 12, 13, 14). On the other hand, the TAF5-TAF6-TAF9-TAF4-TAF12 core is bound by TAF11-TAF13 and TAF3-TAF10 HF pairs and TBP to form lobe A (12, 14). Importantly, *in vitro* the TAF8-TAF10 HF pair does not interact individually with any other HF TAF pair, but it interacts with the 5TAFcore complex, only if all five TAFs of the core complex are simultaneously present and the entire 5TAF core complex is formed (11). In addition, we demonstrated that the building blocks of mammalian TFIID, such as TAF8-TAF10, TAF6-TAF9, and TBP-TAF1, assemble cotranslationally in the cytoplasm, in agreement with the stepwise assembly model of TFIID (15). Early electron microscopy (EM) studies established that endogenous human TFIID resembles a horseshoe composed of three main lobes (16, 17). Recent human and yeast *Komagataella phaffii* TFIID cryoEM structures confirmed the three-lobe structure of TFIID (called lobes A, B, and C) and demonstrated evolutionary conservation and high flexibility within TFIID (12, 14, 18). The high-resolution structures of two TFIID domains indicated that (i) lobe B contains the HFD domains of TAF8-TAF10 histone fold pair, together with one copy of the 5TAF core (TAF5-TAF6-TAF9-TAF4-TAF12) complex, (ii) TAF8 participates in connecting lobe B and C by interacting with the two HEAT repeats of TAF6 and certain regions of TAF1, and (iii) in lobe C the C-terminal half of TAF8 interacts with TAF2 (Fig. 1*A*) (12, 14, 18).

**Figure 1:**
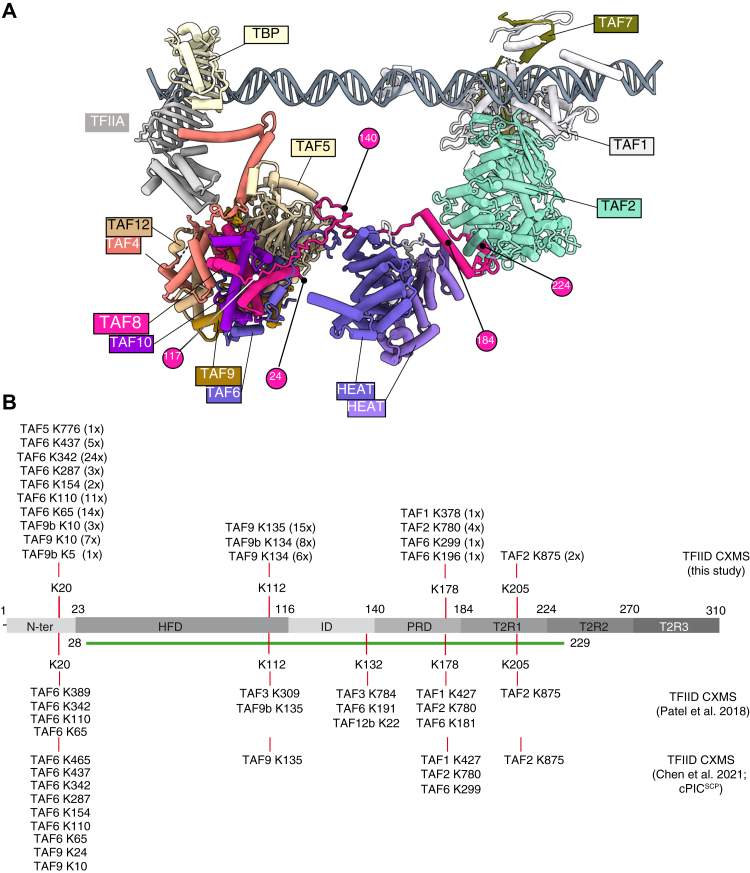
**TAF8 interactions in human TFIID.***A*, the human TFIID cryo-electron microscopy model is shown. The model used in the panel is the super core promoter (SCP)-bound TFIID-TFIIA in post TBP-loading state (12), PDB: 7EGJ. TAF8 is highlighted in *rose*. TAF8 positions used in our study and visible in the TFIID structure are labeled with *rose circles* with their corresponding amino acid positions highlighted in *white*. TBP, TFIIA, and TAFs in the TFIID complex are indicated with their corresponding colors. Lobe B is composed of TAF5, 4–12, 6–9 and TAF8–10, and Lobe C of TAF1, TAF2, and TAF7. The HEAT repeats of the two TAF6s connecting the three lobes of TFIID are highlighted. Lobe A is hidden for clarity (lays behind lobe C from this view). *B*, TAF8 interlinks (cross-links between TAF8 and other TFIID subunits) identified by CXMS analyses of our endogenous TFIID complex preparation. All cross-links were identified with high confidence and the number of times a cross-linked peptide was identified is indicated in *brackets* (Tables S1 and S2). *Upper part*, CXMS done in this study, *lower part*, CXMS done by Patel *et al.* (2018) or Chen *et al.* (2021). Lysines (K) in TAF8 and the residues to which they cross-link on other TAFs are indicated. *Green line* indicates the TAF8 path visible in the cryo-EM structure (12).

In mice, germ line knockout of genes encoding several TFIID subunits (*Taf7*, *Taf8*, *Taf10*, and *Tbp*) results in early embryonic lethality around E4.0 (19, 20, 21, 22), suggesting that these mammalian TFIID subunits are absolutely essential for early mouse development. Furthermore, conditional deletion of either *Taf8* in mouse embryonic stem cells (ESCs) (23) or *Taf10* or *Taf4* in embryonic keratinocytes (24, 25) or deletion of *Taf10* in embryonic liver (26) or ablation of *Taf7* in CD4^−^CD8^−^ thymocytes (20) compromises the viability of all these mutant cells, suggesting that TFIID subunits play essential roles in transcription in the different tested cellular systems.

Importantly, a homozygous *TAF8* TAF8c.781–1G>A splice site mutation in a human patient causes intellectual disability (23). Patients with this mutation express an unstable TAF8 protein in which the C-terminal 49 wild-type amino acids are replaced by a 38 amino acid mutated sequence, caused by the frame shift, leading to partial TFIID dissociation (23). Interestingly, several mutations have also been reported in TAF2 (*i.e.*, T186R, P416H, or W649R), the interaction partner of TAF8, which are also associated with intellectual disability syndrome (27, 28).

In the present study, we have investigated the requirement of different TAF8 regions for TFIID assembly and the importance of some of these TAF8 regions for ESC viability. We have identified TAF8 regions that are either important for interacting with the 5TAF core complex in lobe B or for interacting with TAF2 in lobe C, and we show that the 5TAF core-interacting and that one of the TAF2-interacting TAF8 regions are required for mouse ESC survival.

## Results

### Cross-linking mass spectrometry analysis of human TFIID reveals cross-linking hotspots in TAF8

**Figure 2:**
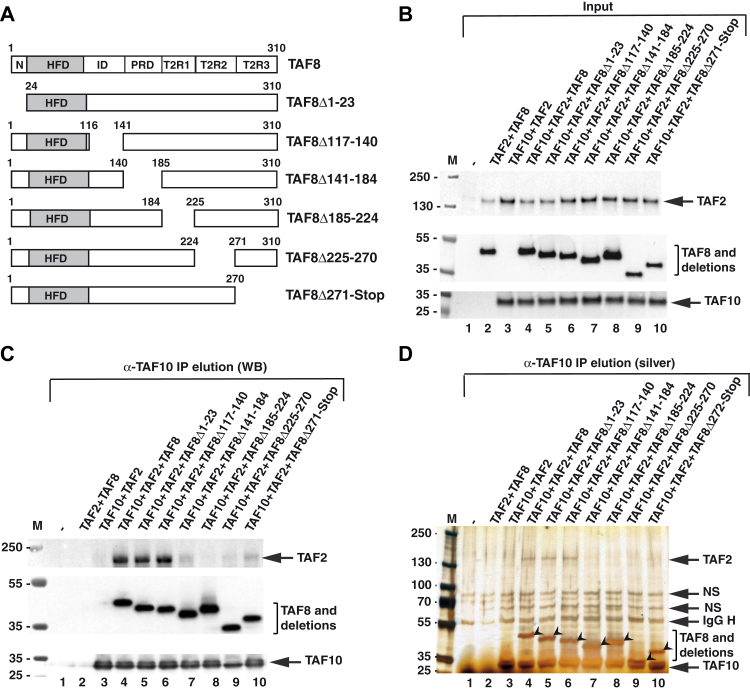
**Characterization of TAF8 interactions within the TAF2-TAF8-TAF10 subcomplex.***A*, schematic presentation of human TAF8, its domain structure, and the different TAF8 deletions. N: N-terminal domain; HFD: histone fold domain; ID: intermediary domain; PRD: proline-rich domain; and T2R1, T2R2, and T2R3: TAF2-interacting regions 1, 2, and 3. *B*–*D*, TAF2 and TAF10 were coexpressed with either WT TAF8 or with TAF8 deletions as indicated above each lane using the Baculovirus overexpression system. Input extracts (*B*) and anti-TAF10 IPs (*C*) were tested by western blot analyses (WB) with the indicted antibodies. *D*, anti-TAF10 IPs were also tested by silver-staining (silver) of the gels. TAF8 and its deletions are indicated with an *arrowhead*. On panels (*B*–*D*) the molecular weight markers (M) are indicated in kDa. *D*, IgG H: heavy chain of the antibody. On panels (*B*–*D*) (lane 1) indicates Sf9 cell extract without any coinfection. IgG H, heavy chain of the antibody; NS, nonspecific band.

To gain more insights into the structure/function relation of human TAF8, first we carried out a multiple sequence alignment of TAF8 proteins from several eukaryotic species to better understand the potential domain conservation of TAF8 in addition to its HFD (Fig. S1). Following our alignment and based on previous publications (9, 10, 11), we have subdivided TAF8 in seven regions: N-ter, HFD, ID (intermediary domain), PRD (proline-rich domain), and T2R1, T2R2, and T2R3 (TAF2-interacting regions 1, 2, and 3) (Figs. 1*A*, 2*A* and S1). Prior to the publication of the cryo-EM structure of hTFIID (14), we performed cross-linking mass spectrometry (CXMS) analyses on TFIID to characterize the architecture of the complex. To this end, we purified endogenous TBP-containing complexes (TFIID, SL1, and TFIIIB) by an anti-TBP immunoprecipitation from HeLa cell nuclear extracts, (Fig. S2), cross-linked them with the amine-reactive cross-linker BS3, and analyzed the sample by CXMS. The CXMS analyses of the endogenous TFIID complex identified 65 interlinks (cross-links between different protein molecules) and 43 intralinks (cross-links involving the same protein) (Tables S1 and S2, respectively). We then focused on TAF8 interlinks where TAF8 cross-linked to another TAF peptide and compared our dataset to CXMS data obtained from super core promoter (SCP)-bound hTFIID or SCP-bound hTFIID containing PIC (12, 14) (Fig. 1*B*). In all of the analyzed endogenous hTFIID complexes, TAF8 cross-linked extensively to TAF2, TAF6, TAF9/9b and less frequently to TAF1, TAF3, TAF5, and TAF12 with cross-linking hotspots in TAF8 at K:20, K:112, K:132, K:178, and K:205 (Fig. 1*B*). The K:20 hotspot of TAF8 cross-links to the TAF9/TAF6 HFD, to the TAF6 middle (TAF6M) and TAF6C domains, containing five HEAT repeats (29), and to one position at the C-terminus of TAF5. These cross-links agree with the potential position of the N-terminal end of TAF8 in the TFIID structure (Fig. 1, *A* and *B*); however, K20 is absent from the published human TFIID structure (PDB: 6MZM, (14)), suggesting that the N-terminal end of TAF8 may be very flexible. K:112 at the C-terminus of the HFD of TAF8 cross-links to a region downstream of the HFD in TAF9/9b and to TAF3. K:132 in the ID of TAF8 cross-links to TAF3, TAF6, and TAF12b. K:178 at the C-terminus of the PRD cross-links to the nonconserved TAF6 linker between TAF6M and TAF6C, to the third HEAT repeat of TAF6, to TAF2 and to the N-terminus region of TAF1. K:205 in T2R1 of TAF8 cross-links in all the three CXMS analyses to K:875 in TAF2. Surprisingly, between amino acids 205 and 310 of TAF8 (situated in T2R2 and T2R3 regions), no cross-linked peptides were detected in any of the three TFIID preparations (Fig. 1*B*), suggesting that either the C-terminal end of TAF8 does not produce peptides that are amenable to MS analysis, it is buried in a non-cross-linkable structure or positioned away from the TFIID surface. Note that the visible path of hTAF8 in the cryo-EM structure of the human TFIID is from amino acids 28 to 229 (PDB: 6MZM, (14)) encompassing the HFD, ID, PRD, and T2R1 regions of TAF8 (Fig. 1*A*). Nevertheless, these cross-linking results in agreement with cryo-EM structures further indicate that TAF8 plays a triple role in TFIID: (i) participates in the assembly of the six HFD-containing 7TAF complex in lobe B *via* its N-terminus and HFD regions; (ii) connects lobes B and C by interacting with the HEAT repeat regions of two TAF6 subunits, the TAF1 region between amino acid (aa) 378 and 427, and TAF2 *via* its PRD; and (iii) interacts with TAF2 in lobe C *via* its C-terminal part.

### Identification of regions of TAF8 that are required either to stably interact with the 5TAF core complex or with TAF2

To further delineate the regions of TAF8 that are important for lobe B and/or lobe C assembly/interactions in TFIID, based on the above determined domain organization (Figs. 1 and S1) and their cross-linking hotspots, we constructed a series of deletion mutants in TAF8. In these deletion mutants, small regions (about 23–45 amino acids) throughout the whole length of human TAF8 were individually deleted, without deleting the HFD of TAF8 to keep the TAF8-TAF10 HF-interaction interface intact (Fig. 2*A*). To evaluate the impact of these deletions on the assembly of recombinant 3TAF (TAF10-TAF8-TAF2), 7TAF (TAF5-TAF6-9/9b-TAF4-12-TAF8-TAF10), and 8TAF (7TAF complex+TAF2) complexes, we used the Baculovirus expression system.

First, we coexpressed TAF10-TAF2 with wild-type (WT) TAF8, or with the six deletion mutants, and carried out TAF10 immunoprecipitations (IPs) (Fig. 2, *B*–*D*), to test whether TAF8 deletion mutants would affect interaction(s) of the TAF8-TAF10 HF pair with TAF2. Our anti-TAF10 coimmunoprecipitation (co-IP) experiments indicated that deletion of PRD (aa 141–184), T2R1 (aa 185–224), T2R2 (aa 225–271), or T2R3 (aa 272-Stop) of TAF8 all abrogated TAF8-TAF10 interactions with TAF2 (Fig. 2, *C* and *D*). Deletions of N-terminal (aa 1–23) and ID (aa 117–140) regions of TAF8 did not influence the formation of the 3TAF (TAF10-TAF8-TAF2) complex (Fig. 2, *C* and *D*). In our negative control experiments, when TAF10 was absent from the coinfections, TAF8 or TAF2 did not copurify by the anti-TAF10 IP, or when TAF8 was absent from the coinfections, TAF10 did not interact with TAF2 (Fig. 2, *B*–*D*, lanes 2 and 3).

**Figure 3:**
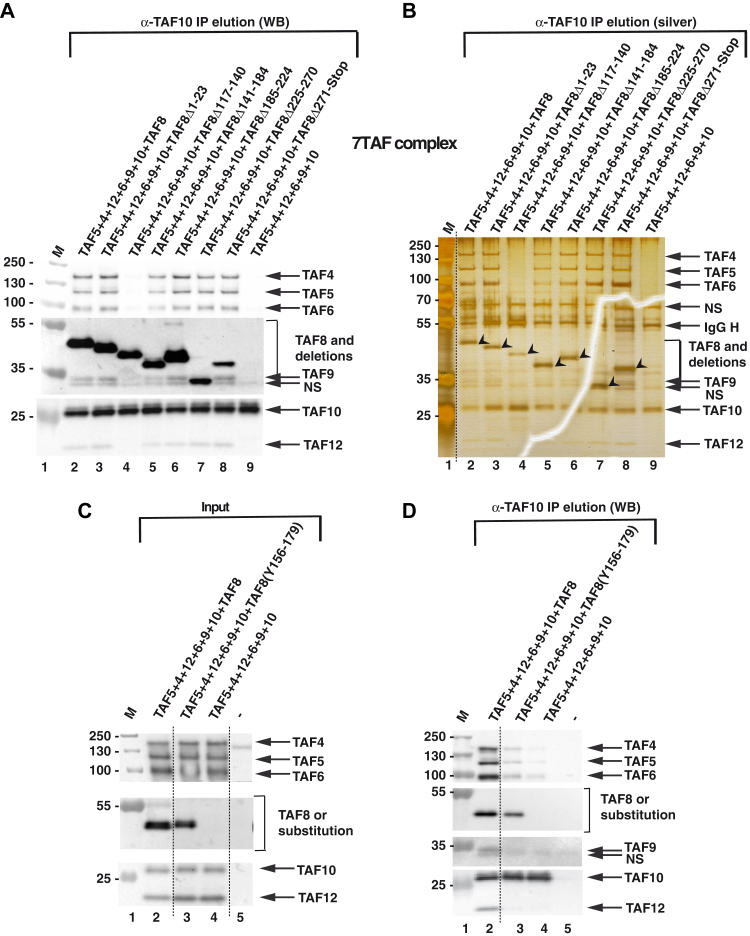
**Characterization of TAF8 interactions within the 7TAF complexes.***A* and *B*, TAF5-TAF6-TAF9-TAF4-TAF12 and TAF10 were coexpressed with either WT TAF8 or with TAF8 deletions as indicated above each lane using the Baculovirus overexpression system. Anti-TAF10 IPs (*A*) were tested by western blot analyses (WB) with the indicted antibodies, or by silver-staining (silver) of the gels (*B*). On panel (*A*) the molecular weight markers (M) are indicated in kDa. *B*, the *arrow heads* indicate TAF8 or the different TAF8 deletions. IgG H: heavy chain of the antibody. The western blot assay tests corresponding to the TAF expressions in the Input extracts for (*A* and *B*) are shown in Fig. S3. *C* and *D*, the substitution of human TAF8 117–140 amino acid sequences with the *S. cerevisiae* Taf8 sequences does not rescue 7TAF complex formation. TAF5-TAF4-TAF12-TAF6-TAF9 and TAF10 were coexpressed with either WT TAF8, or with the TAF8 substitution in which the human 117 to 140 amino acid (aa) sequences were replaced with the yeast 156 to 179 aa sequences ([TAF8(Y156–179)] as indicated above each lane) using the Baculovirus overexpression system. Input extracts (*C*) or anti-TAF10 IPs (*D*) were tested by western blot analyses (WB) with the indicted antibodies. Molecular weight markers (M) are indicated in kDa. -: indicates Sf9 cell extract without any coinfection. *Dotted lines* indicate where the gels were cut. NS, nonspecific band.

Next, subunits of the 7TAF complex were coexpressed with either WT TAF8 or with one of the six deletion mutants (Fig. 2*A*) followed by anti-TAF10 IPs. When analyzing the 7TAF complex formation, we found that only the deletion of the ID region of TAF8 (Δ117–140) severely reduced the interactions with subunits of the 5TAF core complex, while the TAF8-TAF10 HF pair could still form (Figs. 3, *A* and *B* and S2*A*). All other tested TAF8 deletion mutants, including the delta N-terminus mutant deleting the K:20 cross-linking hotspot, which cross-linked extensively to subunits of the 5TAF complex (Fig. 1*B*), formed the 7TAF complex (Fig. 3, *A* and *B*). As described previously (11), TAF10 alone could not interact with the 5TAF core complex when TAF8 was absent from the coinfections, further indicating that the formation of the TAF8-TAF10 HF pair is crucial for forming lobe B complex in TFIID (Fig. 3, *A* and *B*).

To test whether the amino acid sequence of the ID is required for formation of 7TAF complex, we substituted the human TAF8 117–140 amino acid sequences with the *S. cerevisiae* Taf8 sequences from amino acids 156 to 179. TAF10 co-IP results showed that the amino acid substitution in this region could not functionally replace the human sequences, as the 7TAF complex did not form efficiently under these conditions (Fig. 3, *C* and *D*). The results show that the amino acid sequence of the ID domain is required for efficient formation of the 7TAF complex and that the ID is not functionally conserved between *S. cerevisiae* and humans.

**Figure 4:**
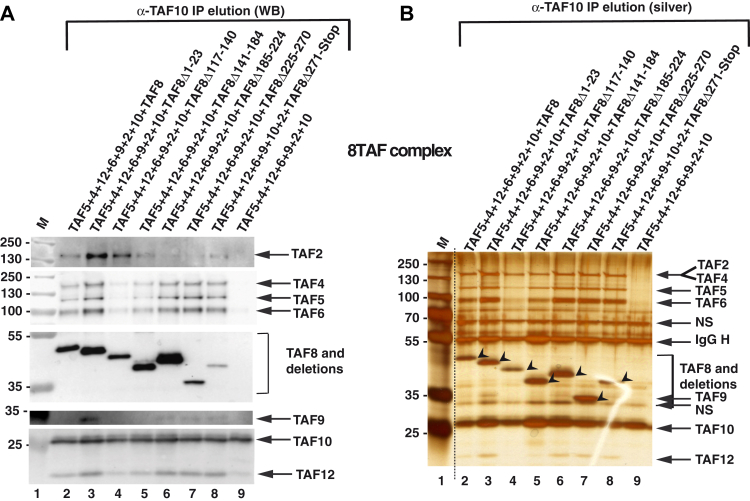
**Characterization of TAF8 interactions within the 8TAF complexes.***A* and *B*, TAF2-TAF5-TAF6-TAF9-TAF4-TAF12 and TAF10 were coexpressed with either WT TAF8 or with TAF8 deletions, as indicated above each lane, using the Baculovirus overexpression system. Anti-TAF10 IP elutions (*A*) were tested by western blot analyses (WB) with the indicted antibodies, or by silver-staining (silver) of the gel (*B*) as described in Figure 3, *A* and *B*. The molecular weight markers (M) are indicated in kDa. *B*, the *arrow heads* indicate TAF8 or the different TAF8 deletions. *Dotted line* indicates where the gel was cut. Input extracts for the anti-TAF10 IPs are shown in Figure S3. IgG H, heavy chain of the antibody; NS, nonspecific band.

When subunits of the 8TAF complex were coexpressed with either WT TAF8 or with one of the six deletion mutants (Figs. 2*A* and S3*B*) and anti-TAF10 IPs carried out, the results recapitulated the observations made individually with either the 3TAF complex or with the 7TAF complex (Figs. 2, *B*–*D* and 3). Namely, in the 8TAF experiments, the TAF8Δ117–140 mutant abrogated the TAF8-TAF10 interactions with the subunits of the 5TAF core complex (TAF5, 4–12, 6–9), but not with TAF2 (Fig. 4, *A* and *B*), and deletion of PRD (Δ141–184), T2R1 (Δ185–224), T2R2 (Δ225–271), or T2R3 (Δ272-Stop) of TAF8 all severely abrogated the TAF8–10 HF pair interactions with TAF2, but did not influence the interactions with the 5TAF core complex (TAF5, 6–9, 4–12,) (Fig. 4, *A* and *B*).

Thus, together our recombinant *in vitro* TFIID subassembly results show that (i) deletion of the nonconserved N-terminal region of TAF8, which cross-links to many core TAF subunits in TFIID (Fig. 1*B*), is not required for formation of the 7TAF core complex, or to interact with TAF2, (ii) the ID region of TAF8 (aa 117–140) is required to interact with the 5TAF core complex, and it cannot be replaced by the ID region from *S. cerevisiae* Taf8, and (iii) PRD (aa 141–184), T2R1 (aa 185–224), T2R2 (aa 225–271), or T2R3 (aa 272-Stop) of TAF8 are all required for stable interactions between TAF8-TAF10 and TAF2.

### Exogenously expressed TAF8 lacking ID, or PRD regions are defective for TFIID assembly in mESCs

**Figure 5:**
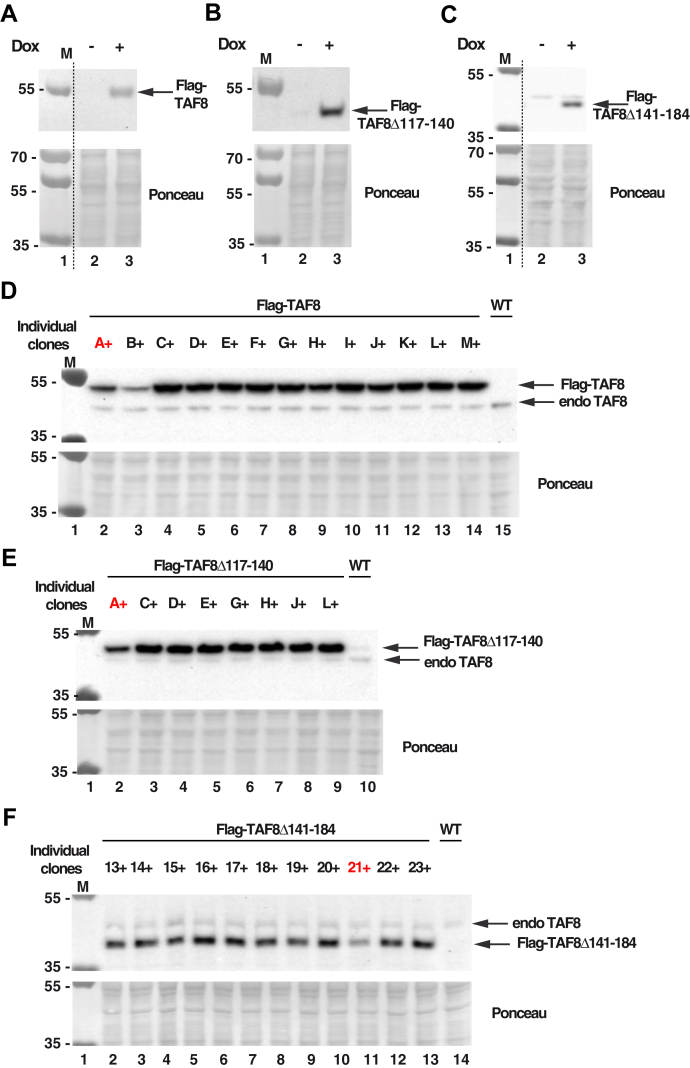
**Generation of ESCs exogenously expressing Flag-TAF8, Flag-TAF8Δ117–140, and Flag-TAF8Δ141–184.***A*–*C*, stable doxycycline (+Dox)-inducible E14 mESC populations were generated in which WT R:Flag-mTAF8 (*A*), R:Flag-mTAF8Δ117–140 (*B*), or R:Flag-mTAF8Δ141–184 (*C*) were exogenously expressed. Stable ESCs were isolated, whole cell extracts (WCE) prepared and tested by western blot assay for the expression of the corresponding Flag-tagged TAF8 proteins using an anti-Flag antibody (as indicated). *D*–*F*, ESC clones originating from one single ESC, expressing the individual Flag-mTAF8 (*D*), Flag-mTAF8Δ117–140 (*E*), or Flag-mTAF8Δ141–184 (*F*) proteins were amplified in presence of Dox (+), WCEs prepared, and the expression of the corresponding TAF8 proteins (as indicated) was tested by western blot assay using an anti-TAF8 antibody. Endogenous (endo) TAF8 is also labeled. Individual ESC clones are labeled A to M (*D*), A to L (*E*) and 13 to 23 (*F*), and the ESC clones selected for further analyses are labeled in *red*. Ponceau staining was used as loading control in all panels. Molecular weight markers (M) are indicated in kDa. *Dotted lines* indicate where the gels were cut in (*A* and *C*).

**Figure 6:**
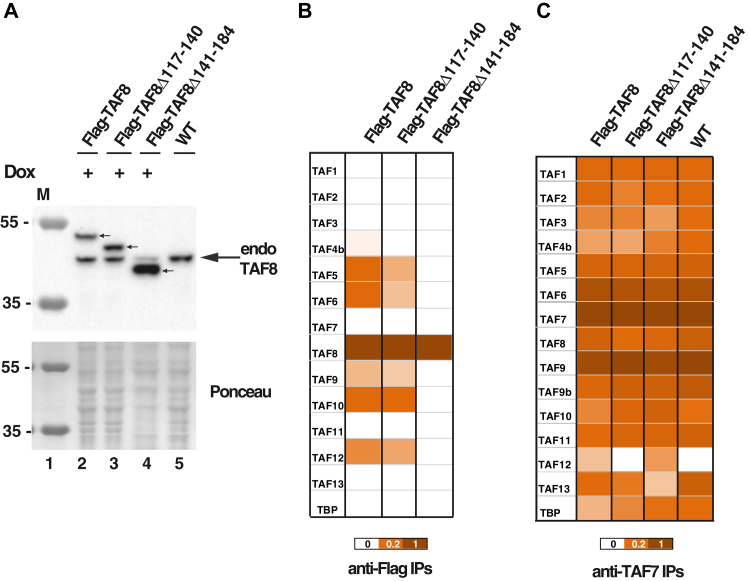
**The exogenously expressed Flag-TAF8Δ117–140 and Flag-TAF8Δ141–184 deletions are impaired in TFIID assembly in mESCs.***A*, exogenously and endogenously expressed TAF8 proteins were compared in the selected Flag-mTAF8(A+), Flag-mTAF8Δ117–140(A+), Flag-mTAF8Δ141–184(21+) clones (see Fig. 5, *D*–*F*) and wild-type (WT) ESC WCEs by western blot analysis using an anti-TAF8 antibody. The different Flag tagged TAF8 proteins are labeled with *small arrows* and the position of endogenous (endo) TAF8 is shown by an *arrow* on the *right*. Ponceau staining was used as loading control in all panels. Molecular weight markers (M) are indicated in kDa. *B* and *C*, mass spectrometry analysis of either anti-Flag (*B*) or anti-TAF7 IPs (*C*) carried out using WCEs (*B*) or nuclear extracts (*C*) prepared from ESCs expressing Flag-TAF8, Flag-mTAF8Δ117–140, Flag-mTAF8Δ141–184, or WT ESCs. Three technical replicates were carried out. Normalized spectral abundance factor (NSAF) values were calculated and normalized to the bait of the IPs (to Flag-tagged TAF8 or its deletions, in *B*; and to TAF7 in *C*). The normalized NSAF results are represented as heat maps with the indicated scales. TFIID subunit identification details are in Table S5.

To test whether the *in vitro* identified regions of TAF8 play a role in TFIID assembly/function in a cellular context, we created mouse ESCs expressing Flag-WT TAF8, Flag-TAF8Δ117–140 (ID), or Flag-TAF8Δ141–184 (PRD) mutants. To generate a Dox-inducible expression system, first we integrated an expression cassette encoding the reverse tetracycline-controlled trans-activator (rtTA; (30)) in E14 ESCs (rtTA-ESC) (Fig. S4). Next we integrated in the genome of the selected rtTA-ESC clone (DD1) a series of cassettes in which the *Flag-WT-TAF8*, *Flag-TAF8Δ117–140*, or *Flag-TAF8Δ141–184* cDNAs are under the control of a tetracycline-operator, resulting in the R:Flag-TAF8WT, R:Flag-TAF8Δ117–140, or R:Flag-TAF8Δ141–184 expressing ESC lines, respectively. The inducibility of Flag-WT-TAF8 protein or its deletions by the tetracycline analogue Dox was verified by western blot analyses using the anti-Flag antibody (Fig. 5, *A*–*C*). Barely any Flag-tagged protein could be detected in cells grown in the absence of Dox, whereas after Dox treatment Flag-TAF8 WT, Flag-TAF8Δ117–140, or Flag-TAF8Δ141–184 mutant proteins were clearly detectable in the corresponding ESC populations (Fig. 5, *A*–*C*). From these ESC populations individual clonal ESC lines, originating from a single cell, were isolated, amplified in the presence of Dox, and tested for the expression of the Flag tagged TAF8 variants *versus* endogenous TAF8 (Fig. 5, *D*–*F*). By selecting the positive ESC clones for further experiments, clones were chosen in which the expression level of each exogenously expressed Flag-TAF8 variants was close to endogenous TAF8 (Fig. 5, *D*–*F*). When we compared the expression levels of the exogenously expressed Flag-WT-TAF8, Flag-TAF8Δ117–140, or Flag-TAF8Δ141–184 proteins in the selected ESC clones, the expression levels of the Flag tagged TAF8 variants (full-length and deletions) were comparable among themselves and also comparable to endogenous TAF8 (Fig. 6*A*).

Next, we set out to analyze whether Flag tagged TAF8 variants (full length and deletions) would incorporate in TFIID, TFIID building blocks and/or would influence endogenous TFIID assembly in mESCs. To this end, we prepared whole cell or nuclear extracts from Dox-treated R:Flag-TAF8 WT, R:Flag-TAF8Δ117–140, or R:Flag-TAF8Δ141–184 expressing cell lines, carried out an anti-Flag (on whole cell extracts) or anti-TAF7 IPs (on nuclear extracts), and subjected the IP-ed complexes to mass spectrometric (MS) analyses. Analyses of the MS data from the Flag IP-ed mESC complexes showed that exogenously expressed WT Flag-TAF8 incorporated in a 7TAF-like complex, indicating that the exogenously expressed TAF8 can form a TFIID assembly intermediate, and that this intermediate resembles the 7TAF complex, containing TAF6–9, TAF4b–12, TAF5, as well as TAF8–10 (Fig. 6*B*). This observation is in agreement with our earlier data, showing that mass spectrometry analysis of an anti-TAF10 IP from WT ESC whole cell extracts does not identify all holo-TFIID subunits, but only a “7TAF-type” complex (31). In contrast to the WT Flag-TAF8 IPs, the Flag-TAF8Δ117–140 mutant's incorporation in such a TAF intermediate complex was somewhat impaired, as the TAF8Δ117–140 (ID) deletion mutant associated with less TAF4B, TAF5, TAF6, and TAF12 (Fig. 6*B*). Surprisingly, under the same IP conditions the Flag-TAF8Δ141–184 (PRD) did not interact with any endogenous TFIID subunits (Fig. 6*B*). Importantly, none of the exogenously expressed Flag-tagged TAF8 proteins (full-length or mutants) impaired endogenous TFIID assembly when nuclear extracts from these cell lines were analyzed by an anti-TAF7 IP (Fig. 6*C*). Thus, these results suggest that the ID or the PRD regions of TAF8 are required for efficient assembly of 7TAF-type complexes (or TFIID subcomplexes) in mESCs.

### TAF8 proteins deleted for ID, or PRD regions do not rescue the survival of *Taf8*^−/−^ ESCs, suggesting that these regions are required for TFIID assembly and function

**Figure 7:**
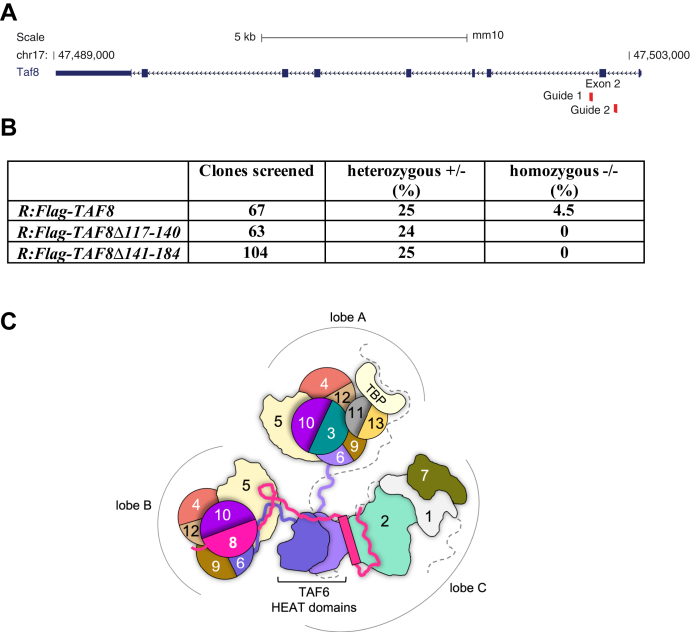
**The expression of Flag-TAF8Δ117–140 or Flag-TAF8Δ141–184 does not rescue the knockout of the endogenous *Taf8* gene in mESCs.***A*, schematic representation of the mouse *Taf8* locus, with exons and introns and with the direction of transcription shown. The genomic positions of the mouse *Taf8* locus on chromosome 17 (chr17) are indicated. The positions of the two gRNAs, surrounding exon 2 used to knockout the endogenous *Taf8* locus, are indicated. *B*, table showing the numbers of individual viable ESC clones (R:Flag-TAF8, R:Flag-TAF8Δ117–140 or R:Flag-TAF8Δ141–184) screened following the CRISPR/Cas9 KO of the endogenous *Taf8* gene. Percentages of the obtained heterozygous (+/−) and homozygous ESC clones (−/−) are indicated. *C*, schematic illustration depicting how TAFs are thought to interact in holo-TFIID (without DNA). The three lobes of TFIID are labeled A to C. TAFs are labeled by their corresponding numbers and the same color code as used Figure 1*A*. HFDs or HFD-containing TAFs are depicted as *circles*. TAF8 regions connecting lobe B and C are shown as a *magenta line* or *cylinder*. Some regions of TAF1 are labeled with a *dotted line*. Domains and connecting lines are not to scale.

To test whether the above identified ID or PRD regions of TAF8 are required for *in vivo* TFIID assembly and consequent mESC survival, we set out to inactivate the endogenous *Taf8* gene using CRISPR/Cas9 genome editing in the ESC lines expressing R:Flag-TAF8 WT, R:Flag-TAF8Δ117–140 (ID), or R:Flag-TAF8Δ141–184 (PRD) (Fig. 7*A*). Using this strategy in the presence of Dox in the R:Flag-TAF8 WT mESC expressing line, we obtained several viable homozygous *Taf8* deletion ESC lines (Fig. 7*B*), indicating that the CRISPR/Cas9 worked efficiently. By using the same gene editing strategy in the presence of Dox in R:Flag-TAF8Δ117–140 or R:Flag-TAF8Δ141–184 expressing cell lines, we obtained heterozygous clones in the same proportion as for the Flag-TAF8 WT expressing lines, indicating that the CRISPR/Cas9 worked with comparable efficiency at the *Taf8* genomic locus in these heterozygous mESC lines, as in the R:Flag-TAF8 WT mESC expressing line (Fig. 7*B*). In contrast, we could not isolate any homozygous knockout clones when inactivating the *Taf8* locus in the R:Flag-TAF8Δ117–140 or in the R:Flag-TAF8Δ141–184 mESC lines, suggesting that the deleted ID or PRD regions of TAF8 have essential roles in endogenous TFIID assembly and/or function, and consequently for mESC survival, and that these functions cannot be compensated by other TAFs or TAF assemblies.

## Discussion

Characterization of the structure–function relationship of multisubunit transcription complexes is crucial to understand gene regulation. Whereas three-dimensional models of fully assembled multiprotein complexes derived mainly from single-particle cryo-electron microscopy provide a wealth of information on the architecture of such complexes, the requirement of the precise subunit–subunit and domain–domain interactions to maintain the architecture and function of these gene regulatory complexes, such as TFIID, remains largely elusive.

Based on several functional and structural studies (9, 10, 14, 18, 19, 32), as well as on three independent CXMS experiments carried out with endogenous TFIID (this study and (12, 14)), we have subdivided TAF8 into seven regions (Fig. 2*A*). Out of these seven regions, the HFD of TAF8 has already been characterized (10); thus we have investigated how the deletion of the remaining six regions of TAF8 influences the *in vitro* assembly of TAF8-containing TFIID building blocks. We identified three types of regions: (i) the N-terminus of TAF8 that did not disrupt the tested interactions with either the 5TAFcore complex or with TAF2, (ii) the ID region that is required for interactions with the 5TAF core complex, but does not influence the TAF2 interactions, and (iii) four successive TAF2 regions, which are all required for normal TAF8-TAF2 interactions, but do not influence the interactions with the 5TAF core complex (Figs. 2, 3 and 7*C*). Our TAF8-TAF2 interaction results agree with previous surface plasmon resonance (SPR) experiments using immobilized full-length TAF2 as ligand and maltose-binding protein (MBP) fusions of TAF8 fragments (10). Thus, our deletion analysis confirms that TAF8 plays a triple role in TFIID: the HFD together with the ID region of TAF8 participate in the assembly of the six HFD-containing 7TAF complex in lobe B. Importantly, when the ID region of human TAF8 was replaced with the nonconserved amino acids from yeast TAF8, this replacement did not restore the binding of the TAF8-TAF10 HF pair to the 5TAF core complex, further substantiating that this region and its amino sequence are important for forming the 7TAF complex in lobe B. It is possible that residues within the ID make specific contacts with TAF8, or TAF6, or other components of the 5TAF complex that are required for proper interaction(s) of TAF8-TAF10 with the 5TAF complex. In spite of the fact that TAF8 PRD cross-links to the TAF6 HEAT repeat regions, deletion of TAF8 PRD (aa 141–184) did not influence the formation of the 7TAF complex, suggesting that the TAF8 PRD is involved in the lobe B-C connecting function of TAF8, but not important for lobe B formation. In the cryo-EM structures of TFIID, the lobe B-C connecting function of TAF8 was proposed to involve PRD. Importantly, PRD is one of the most conserved regions of TAF8 (Fig. S1), suggesting that this domain is involved in well-conserved structural interactions with TAF2. Our deletion mutant analyses could not separate the lobe B-C connecting function of TAF8 from its TAF2 interacting function. Previous pairwise biophysical interaction assays between TAF8 and TAF2 suggested that the TAF8 region spanning from amino acid 141 to amino acid 200 (overlapping PRD, defined in this study) is important for interactions with TAF2 (10). The fact that only TAF8 and TAF2 were tested in these assays would argue that the well-conserved PRD would rather form structural interactions with TAF2, than a linker-like interaction, since no other lobe C proteins were present in these interaction studies. In addition, the recent cryo-EM structures of TFIID show that the C terminal region of the PRD (aa 171–184) is part of a helix that bridges the heat repeat of the second copy of TAF6 and TAF2 (12, 14). Furthermore, we found that the deletion of either the PRD or each of the three TAF2 interaction regions (T2R1-T2R3 mutants) individually all abrogated the interaction of TAF8 with TAF2. Our present deletion experiments, together with the above-mentioned SPR or cryo-EM studies, suggest that the entire 170 amino acid TAF8 tail is needed to interact with TAF2 (Fig. 7*C*). These interactions could happen through several TAF8-TAF2 contact surfaces, which would be all synergistically required for connecting lobes B and C. In the cryo-EM structures of TFIID, the structured TAF8 path stops at amino acid 229 (Fig. 1*A*) (14), leaving about 100 aa of TAF8 unresolved. Similarly, the unresolved regions of TAF2 in the cryo-EM structure could also be involved in TAF8 interactions. Nevertheless, no TAF8-TAF2 cross-links have been detected in endogenous TFIID complexes either in T2R2 or T2R3 regions. In this respect, it is interesting to note that the deletion of the T2R2 or T2R3 regions of TAF8 abrogates the TAF2 interactions, suggesting that these regions are still crucial for TAF2 interactions. Intriguingly, the patient with intellectual disability expresses an unstable TAF8 protein in which the C-terminal last 49 WT amino acids of TAF8 are replaced by a 38 amino acid mutated sequence (which removes part of T2R2 and all of T2R3), caused by the frame shift, leading to TFIID dissociation (23). In addition, the reported intellectual disability causing TAF2 mutations (T186R, P416H, or W649R) may also fall in these unmapped TAF2-TAF8 interaction regions (27, 28). It is thus conceivable that an important regulatory surveillance mechanism exists in cells to control TAF8-TAF2 interactions and through these interactions stable holo-TFIID assembly. Nevertheless, pluripotent ESCs with highly active Pol II transcription seem to require fully assembled and functional holo-TFIID as deletion of *Tbp*, *Taf7*, *Taf8*, and *Taf10* causes mESC lethality (19, 20, 21, 22, 23). Our results suggest that when the ability of TAF8 to interact with the 5TAF core complex in lobe B (TAF8ΔID) or with TAF2 in lobe C (TAF8ΔPRD) is impaired, likely causing defective TFIID assembly, ESCs cannot survive, further suggesting that ESCs require fully assembled holo-TFIID for function. Astonishingly, mutant TAF8 patient fibroblasts in which no TAF8 could be detected presented no detectable Pol II transcription defects (23), and in adult mouse keratinocytes deletion of *Taf10* had no effect on transcription and epidermal function even after wounding or UV irradiation (24). Also, mouse CD4+CD8+ T-cells lacking *Taf7* are viable with no apparent effect on transcription (20). Thus, it seems that certain cell types, mainly at later developmental stages, do not require holo-TFIID for Pol II transcription and cell survival, while other cell types, such as pluripotent ESCs, are absolutely dependent on holo-TFIID assembly and function. Future experiments will determine whether deletions of ID or PRD regions of TAF8 at later stages of ESC differentiation (*i.e.*, embryonic body or neuronal differentiation) would also cause cell death.

Here we have identified regions of TAF8 that are required for (1) anchoring TAF8-TAF10 to the core 5TAF complex and (2) for connecting the 7TAF complex in Lobe B to Lobe C (Fig. 7*C*). TAF8 plays a critical role in the genesis of TFIID and mutations in TAF8 can lead to human diseases such as ID. As such, defining the role of TAF8 regions in TFIID assembly and function provides key information about how TFIID assembly occurs and how mutations in a particular domain can lead to human diseases. Altogether, we show the significance of precise interactions of TAF8 with other TAFs, establishing TAF8 as a functional bridge between lobes B and C in TFIID (Fig. 7*C*). Moreover, our experiments indicate that these interactions in TFIID are required for mESCs to function properly.

## Experimental procedures

### Plasmids

The Baculovirus expression vector for FLAG-tagged human (h) TAF8 WT was PCR amplified from the pPBAC MultiBac vector, expressing TAF8 and TAF10 together, described in (10), and the cDNA was inserted in the pVL1393 Baculovirus expression vector digested with Bam HI and Eco RI together with a primer coding for a Flag-tag on the 5′ end of the hTAF8 cDNA. The TAF8 deletion series was generated by site-directed mutagenesis using pVL1393-Flag-TAF8 WT vector as a template (see Fig. 2*A*). The deleted Flag-TAF8 cassettes were inserted into the pVL1393 Baculovirus expression vector. The constructs were verified by sequencing. All the other Baculovirus expression vectors have been described previously (11, 33).

Mouse (m) WT Flag-TAF8 cDNA and its deletions, corresponding to the hTAF8 deletions (called Flag-mTAF8Δ117–140 or Flag-mTAF8Δ141–184), were cloned into the pUHD10-3 vector (34) in which the expression of WT Flag-mTAF8, Flag-mTAF8Δ117–140, or Flag-mTAF8Δ141–184 is under the control of tet-operator.

The pUES-3 plasmid expressing the two *Taf8* guide (g) RNAs and coexpressing high-fidelity Cas9 (35) fused to EGFP (Cas9-HF-EGFP) were generated by Golden Gate cloning (36). The sequences of the gRNAs are shown in Table S3. The pUES-3 plasmid was verified by sequencing.

### Antibodies

Mouse monoclonal (mAb) and rabbit polyclonal (pAb) antibodies raised against the following proteins have been described previously or were purchased commercially: anti-TBP (mAb 3G3) (37) and (mAb 2C1) (38), anti-TAF2 (pAb 3083) (10), anti-TAF4 (mAb 32TA2B9) (22), anti-TAF5 (mAb 1TA1C2) (39), anti-TAF6 (mAb 25TA2G7) (39), anti-TAF7 (mAb 19TA) (40) and (pAb 3475) (31), anti-TAF8 (pAb 3478) (31), anti-TAF10 (mAb 6TA2B11) (41) and TAF12 (22TA) (42), anti-VP16 mAbs 2GV4, 5GV7 and 5GV2 (43), anti-γ-Tubulin (Sigma Aldrich T6557), and anti-FLAG [M2 antibody (F3165 Sigma-Aldrich)].

### Mouse ESC culture conditions

Mouse E14 ESCs were cultured on plates coated with 0.1% gelatin solution in 1× PBS (Dutcher, Cat# P06-20410) using DMEM (4.5 g/l glucose) with 2 mM Glutamax-I medium supplemented with 15% fetal calf serum (FCS) (Thermo Fisher Scientific, Cat# 10270-106), 0.1% β-mercaptoethanol (Thermo Fisher Scientific, Cat# 31350-010), 40 μg/ml gentamicin (KALYS, Cat# G0124-25), 0.1 mM nonessential amino acids (Thermo Fisher Scientific, Cat# 11140-035). 1.500 U/ml leukemia inhibitory factor (LIF) (home-made), 3 μM CHIR99021 (Axon Medchem, Cat# 1386) together with 1 μM PD0325901 (Axon Medchem, Cat# 1408) were added freshly to the medium. Cells were grown at 37 °C with 5% CO_2_. For cell collection and amplification, ESCs were trypsinized for 2 to 3 min with trypsin-EDTA (Invitrogen, Cat# 25200-072) and the digestion was stopped by the addition of prewarmed 15% FCS. Where indicated doxycycline (Dox) was added to the medium at a final concentration of 1 μg/ml (Sigma Cat#D9891).

### Generation of conditional *Taf8* mutant mouse embryonic stem cells

To obtain the mESC line stably expressing the reverse tetracycline activator rtTA, first E14 mESCs were transfected with the pDG1-rtTA plasmid (Aat II linearized), together with 1/10 of pKJ1B plasmid (for neomycin G-418 selection; Hind III linearized) as described previously (30, 44), using Lipofectamine 2000 (Thermo Fisher Scientific, Cat# 11668-019). To select E14 mESCs clones stably expressing rTA, first genomic DNA was extracted and genomic PCR was carried out with primers QY74 and WK31 (Table S4). Next, RNA was prepared with NucleoSpin^R^ RNA mini isolation kit (MACHEREY-NAGEL, GmbH&Co.KG, Cat# 740955), and RT-qPCR was carried out using HA273 and HA274 primers for detecting *rtTA* expression, and HA817 and HA818 primers for *Rplp0* housekeeping gene amplification (Table S4), which was used for normalization. The expression of the rtTA protein was verified by western blot analyses using anti-VP16 mAbs 2GV4, 5GV7 and 5GV2 (43).

Next the rtTA expressing ESC line was transfected using Lipofectamine2000 (Thermo Fisher Scientific, Cat# 11668-019), with pUHD-Flag-mTAF8, pUHD-Flag-mTAF8Δ117–140, or pUHD-Flag-mTAF8 Δ141–184 plasmids (each Hind III linearized), in which the expression of full-length mTAF8 and the two mTAF8 deletion mutants was under the control of tet-operator, together with 1/10 of pGK Hygro plasmid (for hygromycin selection; Pvu II linearized). Hygromycin (Sigma Cat#HO654) selection (250 μg/ml) was started 2 days after transfection (44). Single cell derived colonies were recovered 8 days after hygromycin selection and further amplified in 3 cm wells. After inducing the expression of the different *Taf8* transgenes for 3 days with 1 μg/ml doxycycline (Dox, D9891 Sigma-Aldrich), positive ESC clones were selected and analyzed by western blot assays using an anti-Flag mouse monoclonal antibody or purified anti-TAF8 rabbit polyclonal antibody (pAb3478).

### Knocking out the endogenous Taf8 locus

The E14 derived R:Flag-mTAF8, R:Flag-mTAF8 Δ117–140, and R:Flag-mTAF8 Δ141–184 clonal ESC lines at a confluency of 70 to 80% (2 × 10^6^ cells on a 10 cm Petri dish) were transfected with the pUES-3 plasmid (24 μg), expressing the two *Taf8 gRNAs* (Fig. 7*A* and Table S3) and the Cas9-GFP fusion protein, using Lipofectamine 2000 (Thermo Fisher Scientific, Cat# 11668-019). Dox was omitted during transfection and was added 6 h after transfection. Two days later, the transfected R:Flag-mTAF8, R:Flag-mTAF8 Δ117–140, and R:Flag-mTAF8 Δ141–184 cells were selected based on the detection of the Cas9-GFP fusion protein by fluorescence activated cell sorting (FACS). Five 96-well plates were seeded with one GFP-positive cell per well using the BD Biosciences FACSAria Fusion apparatus equipped with Biosafety Cabinet, and cells were further cultured for 8 days, media was changed every 2 days. Mouse ESC clones originating from a single transfected ESC were collected, transferred to 48-well plates, amplified in the presence of Dox, and 1/5th of the cells were used for PCR genotyping. Genomic DNA was extracted, and PCR carried out using primers described in Table S4 with the Phire direct PCR kit (Thermo Scientific, Cat# F-1265; following manufacturer’s instructions).

### RT-qPCR

The isolated RNA samples were reverse transcribed to cDNA using superscript II (Transcriptase inverse SuperScript II, Invitrogen Cat# 18064022) following manufacturer’s instructions. Then the cDNA samples were amplified using LightCycler 480 SYBR Green 2× PCR Master Mix I (Roche, Cat# 04887352001) and 0.6 μM of forward and reverse primers with a LightCycler 480 (Roche). The primer pairs used for different RT-qPCR reactions are listed in Table S4. For the assessment of mRNA levels, the obtained threshold values were used to calculate the relative gene expression using the 2-ΔΔCT method and considering the individual primer pair efficiencies (45).

### Whole cell extract preparations

The required number of cells were trypsinized, transferred to 1.5 ml Eppendorf tubes, centrifuged at 600*g* 4 °C for 2 min, and washed once with 1 ml 1× PBS. Pellets were resuspended in one packed cell volume (PCV) extraction buffer (600 mM KCl, 50 mM Tris-HCl pH 7.9, 25% glycerol, 0.2 mM EDTA, 0.5 mM DTT, 5 mM MgCl_2_, 0.5% NP40, 1× protease inhibitor cocktail), incubated 5 to 10 min on ice, and mixed with three PCV of “IP0 buffer” (25 mM Tris-HCl pH 7.9, 5% glycerol, 1 mM DTT, 5 mM MgCl_2_, 0.1% NP40, 1× protease inhibitor cocktail). After further 10 min incubation on ice, tubes were centrifuged at 14,000*g* at 4 °C for 10 min. The supernatant fractions, called whole cell extracts (WCEs), were stored at −80 °C.

### Recombinant protein production

Recombinant Baculoviruses were generated as described (10); Demeny, 2007 #3366} and used for protein complex production in *Sf9* insect cell culture. Infected insect cells were harvested 48 h post cell infection by centrifugation and stored at −80 °C until further use. Pellets of Baculovirus-infected *Sf9* insect cells, coexpressing the different TAFs and distinct TAF8 proteins (as indicated in the Figure 2, Figure 3, Figure 4), were resuspended in lysis buffer (400 mM KCl, 50 mM Tris-HCl pH 7.9, 10% glycerol, 0.2 mM EDTA, 0.5 mM DTT, containing 1× protease inhibitor cocktail). Extracts were prepared by three rounds of freeze–thawing and clearing by centrifugation. The supernatant fractions were stored at −80 °C.

### Immunoprecipitation experiments

Protein-G (Protein G Sepharose 4 Fast, cat# GE17-0618-01) or Protein-A (ProteinA-Sepharose 4, cat#P9424 Millipore) beads were washed twice with 1× PBS and twice with IP100 buffer (25 mM Tris-HCl 7.9, 5 mM MgCl_2_, 10% glycerol, 0.1% NP40, 100 mM KCl, 2 mM DTT, and 1× protein inhibitor cocktail). Starting input protein extracts were either ESC nuclear extracts (0.5–2 mg), ESC WCEs (0.5–2 mg), or Baculovirus-infected Sf9 cell extracts (2–5 mg). If needed, protein extracts were diluted with “0” buffer (25 mM Tris-HCl pH 7.5, 5 mM MgCl_2_, 10% glycerol, 0.1% NP40, 1 mM DTT, and 1× protease inhibitor cocktail) to reach a final 100 mM KCl concentration in the extracts. Protein inputs were then precleared by the addition of 1/10 volume of 100% protein A or G beads for 1 h at 4 °C with overhead agitation. During this time beads were coupled to the different antibodies (as indicated in the figure legends). Approximately, 1 mg of indicated antibody per ml of protein A or G bead was bound. Beads were incubated with the antibodies for 1 h at room temperature with agitation, unbound antibody was removed by washing the beads twice with IP500 buffer (“0” buffer containing 500 mM KCl) and twice with IP100 buffer before addition of the precleared protein extracts, and further incubated overnight at 4 °C with overhead agitation. The following day the beads were collected, and subjected to two rounds of washing for 10 min each with ten volumes of IP500 buffer, followed by 2× IP100 buffer washes. Proteins IP-ed with an anti-TBP mAb were eluted by adding one bead volume of 2 mg/ml competing epitope peptide for 4 h, repeated again for 2 h (41). For anti-Flag and anti-TAF7 IPs, proteins were eluted with 0.1 M pH 2.8 glycine, then neutralized with 1.5 M Tris pH 8.8. Eluted proteins were either separated on a 4 to 12% SDS-PAGE gel, along with the input extract, and were probed with antibodies as indicated in the different figures, silver-stained, or analyzed by mass spectrometry.

### Chemical cross-linking mass spectrometry

In total, 1.2 ml anti-TBP immunopurified proteins (∼1.14 mg) were cross-linked by addition of BS3 (Thermo Scientific; freshly prepared as 100 mM in pure water) to 2 mM for 2 h at 25 °C. The reaction was quenched by addition of 10 μl of 1 M ammonium bicarbonate. Then 20 μl SP3 beads (20 μg/μl) were added, as described (46), followed by an equal volume of acetonitrile, and the sample was incubated at 60 °C for 30 min with shaking. Then the beads were concentrated with a magnet and washed with 100% acetonitrile. The beads were then suspended in 100 μl 8 M Urea in 1 M ammonium bicarbonate and treated with TECP/IAA for 2 h at 37 °C in the dark. Then the samples were diluted ten times with water and digested by addition of trypsin [20:1 (w/w), protein:trypsin] overnight at 37 °C. The peptide sample was desalted by passage over 1 cc C18 cartridges (Waters), and dried by Speed-Vac. The peptides were resuspended in 50 μl Buffer A (25 mM ammonium formate, 20% acetonitrile, 0.1% formic acid, pH 2.8). One microgram of each sample was reserved for direct MS analysis, and the remaining sample was fractionated using an in-house prepared microcapillary strong cation exchange column (Proteomix SCX 3um, Sepax Technologies). We used a binary HPLC pump with split flow with microcapillary flowrate at 2 to 3 μl/min. Peptides were loaded onto the microcapillary column equilibrated in Buffer A and washed with Buffer A. Bound peptides were eluted with 20 μl of Buffer A containing 50% and 85% Buffer B (800 mM ammonium formate, 20% acetonitrile, pH 2.8), followed by 50 μl elutions with Buffer B containing 5% of Buffer D (0.5 M ammonium acetate, 30% acetonitrile), or 20 μl of 100% Buffer D. All fractions were dried in a Speed-vac and resuspended in 0.1% trifluoroacetic acid (TFA), 2% acetonitrile. Peptides were analyzed by electrospray ionization microcapillary reverse-phase HPLC on a Thermo Scientific Fusion with HCD fragmentation and serial MS events that included one FTMS1 event at 30,000 resolution followed by FTMS2 events at 15,000 resolution. Other instrument settings included: MS1 scan range (m/z): 400 to 1500; cycle time 3 s; Charge states 4 to 10; Filters MIPS on, relax restriction = true; Dynamic exclusion enabled: repeat count 1, exclusion duration 30 s; Filter Intensity Threshold, signal intensity 50,000; Isolation mode, quadrupole; Isolation window 2 Da; HCD normalized collision energy 28%, isolation width 2 Da; AGC target 500,000, Max injection time 200 ms. A 90 min gradient from 5% ACN to 40% ACN was used.

The RAW files were converted to mzXML files by Rawconverter (47). For cross-linked peptide searches, we used two different cross-link database searching algorithms: pLink (48) and an in-house designed Nexus. Cross-linking data were analyzed using pLink (48) with default settings (precursor monoisotopic mass tolerance: ±10 ppm; fragment mass tolerance: ±20 ppm; up to four isotopic peaks; max evalue 1; static modification on Cysteines; 57. 0215 Da; differential oxidation modification on Methionines; 15.9949 Da) against a database containing TFIID, TFIIIB, and SL1/TIF-IB protein sequences. The Nexus program can be directly downloaded from https://www.dropbox.com/sh/o7z1h12sf3nu89f/AAD5tR_iEXaf8IUDcYZjSj3ja?dl=0. For Nexus searches, the same databases were used with the following parameter settings: (a) up to three miscleavages; (b) static modification on Cysteines (+57.0215 Da); (c) differential oxidation modification on Methionines (+15.9949 Da); (d) differential modification on the peptide N-terminal Glutamic acid residues (−18.0106 Da) or N-terminal Glutamine residues (−17.0265 Da); (e) differential mono-BS3 modification on Lysine residue (+156.0806 Da). A 5% of FDR cutoff was used for both pLink and Nexus. After performing the pLink and Nexus analyses, the search results were combined and each spectrum was manually evaluated for the quality of the match to each peptide using the COMET/Lorikeet Spectrum Viewer (TPP). Cross-linked peptides are considered confidently identified if at least four consecutive b or y ions for each peptide are observed and the majority of the observed ions are accounted for. Search results that did not meet these criteria were removed. Intralinks involving a cross-link between identical residues were only kept if the spectral evidence strongly supported the identification; that is, the major fragment ions correspond to the intralinked peptide sequence and no/few other fragment ions were observed. All the cross-linked spectra and cross-linking maps can be viewed at: https://www.yeastrc.org/proxl_public/viewProject.do?project_id=153.

### LC MS/MS mass spectrometry analyses

Protein mixtures were precipitated with TCA (Sigma Aldrich, Cat# T0699) overnight at 4 °C. Samples were then centrifuged at 14,000*g* for 30 min at 4 °C. Pellets were washed twice with 1 ml cold acetone and centrifuged at 14,000*g* for 10 min at 4 °C. Washed pellet were then urea-denatured with 8 M urea (Sigma Aldrich, Cat# U0631) in Tris-HCl 0.1 mM, reduced with 5 mM TCEP for 30 min, and then alkylated with 10 mM iodoacetamide (Sigma Aldrich, Cat# I1149) for 30 min in the dark. Both reduction and alkylation were performed at room temperature and under agitation (850 rpm). Double digestion was performed with endoproteinase Lys-C (Wako, Cat# 125-05061) at a ratio 1/100 (enzyme/proteins) in 8 M urea for 4 h, followed by an overnight modified trypsin digestion (Promega, CAT# V5113) at a ratio 1/100 (enzyme/proteins) in 2 M urea for 12 h.

Samples were analyzed using an Ultimate 3000 nano-RSLC (Thermo Fisher Scientific) coupled in line with a LTQ-Orbitrap ELITE mass spectrometer *via* a nano-electrospray ionization source (Thermo Fisher Scientific). Peptide mixtures were loaded on a C18 Acclaim PepMap100 trap-column (75 μm ID × 2 cm, 3 μm, 100 Å, Thermo Fisher Scientific) for 3.5 min at 5 μl/min with 2% ACN (Sigma Aldrich, Cat# 1207802), 0.1% formic acid (Sigma Aldrich, Cat# 94318) in water and then separated on a C18 Accucore nano-column (75 μm ID × 50 cm, 2.6 μm, 150 Å, Thermo Fisher Scientific) with a 90 min linear gradient from 5% to 35% buffer B (A: 0.1% FA in water/B: 99% ACN, 0.1% FA in water), then a 20 min linear gradient from 35% to 80% buffer B, followed with 5 min at 99% B and 5 min of regeneration at 5% B. The total duration was set to 120 min at a flow rate of 200 nl/min. The oven temperature was kept constant at 38 °C. The mass spectrometer was operated in positive ionization mode, in data-dependent mode with survey scans from m/z 350 to 1500 acquired in the Orbitrap at a resolution of 120,000 at m/z 400. The 20 most intense peaks (TOP20) from survey scans were selected for further fragmentation in the Linear Ion Trap with an isolation window of 2.0 Da and were fragmented by CID with normalized collision energy of 35%. Unassigned and single charged states were rejected. The Ion Target Value for the survey scans (in the Orbitrap) and the MS2 mode (in the Linear Ion Trap) were set to 1E6 and 5E3, respectively, and the maximum injection time was set to 100 ms for both scan modes. Dynamic exclusion was used. Exclusion duration was set to 20 s, repeat count was set to 1, and exclusion mass width was ±10 ppm. TFIID subunit identification details are shown in Table S5.

#### Data analysis

Proteins were identified by database searching using SequestHT (Thermo Fisher Scientific) with Proteome Discoverer 2.4 software (PD2.4, Thermo Fisher Scientific) on *Mus musculus* database (Swissprot, nonreviewed, release 2019_08_07, 55,121 entries). Precursor and fragment mass tolerances were set at 7 ppm and 0.6 Da, respectively. Trypsin [KR/P specificity] was set as enzyme and up to two missed cleavages were allowed. Oxidation was set as variable modification, and carbamidomethylation as fixed modification. Peptides were filtered with a false discovery rate (FDR; which was calculated based on decoy-reverse database used by the Proteome Discoverer 2.4 software) at 1%, rank 1, and proteins were identified with 5% FDR and one unique peptide. For the Label-Free Quantification, the protein abundancies were calculated from the average of the peptide abundancies using the TOP N (where N = 3, the three most intense peptides for each protein), and only the unique peptide was used for the quantification. Data were analyzed by calculation of NSAF (31, 49).

## Data availability

The proteomics data have been deposited to the ProteomeXchange Consortium *via* the PRIDE (50) partner repository, the CXMS data with the dataset identifier PXD026575 and the affinity-based LC MS/MS data with the dataset identifier PXD028352.

## Supporting information

This article contains supporting information.

## Conflict of interest

The authors declare that they have no conflicts of interest with the contents of this article.
